# Human Cellulosimicrobium Infections: A Clinically Oriented Review of Diagnostic and Therapeutic Challenges

**DOI:** 10.7759/cureus.113612

**Published:** 2026-07-29

**Authors:** Elmostafa Benaissa, Najwa Hnach, Yassine Ben Lahlou, Mariama Chadli

**Affiliations:** 1 Bacteriology, Mohammed V Military Training Hospital, Rabat, MAR; 2 Clinical Bacteriology, Mohammed V Military Training Hospital, Rabat, MAR; 3 Medicine, Mohammed V University, Rabat, MAR

**Keywords:** bacteremia, cellulosimicrobium cellulans, infective endocarditis, maldi-tof ms, oerskovia xanthineolytica

## Abstract

*Cellulosimicrobium *species, formerly classified within the genus *Oerskovia*, are environmental Gram-positive filamentous bacilli that rarely cause human infection. Their clinical significance may be underestimated because coryneform Gram-positive rods are frequently interpreted as contaminants in routine cultures. Publications on human infections are dominated by case reports and small case series, most often involving bloodstream infection, infective endocarditis, osteoarticular infection, peritoneal dialysis-associated peritonitis, and endophthalmitis. Reported cases commonly occur in patients with immunosuppression, malignancy, renal replacement therapy, prosthetic cardiac valves, central venous catheters, or other foreign material. *Cellulosimicrobium cellulans *and its former name *Oerskovia xanthineolytica* account for most described infections. Diagnostic confirmation usually requires careful interpretation of repeated positive cultures, isolation from sterile sites, and reliable identification using matrix-assisted laser desorption ionization-time-of-flight mass spectrometry or 16S ribosomal ribonucleic acid gene sequencing when available. Therapeutic evidence remains limited, but vancomycin and trimethoprim-sulfamethoxazole generally retain in vitro activity, whereas resistance to beta-lactams and clindamycin has been reported. Management should combine species-level identification, susceptibility testing, appropriate antimicrobial therapy, and source control, including removal of infected devices whenever feasible.

## Introduction and background

The genus *Cellulosimicrobium* comprises Gram-positive actinobacterial rods belonging to the family Promicromonosporaceae. These organisms are widely distributed in soil, sewage, water, and plant material. In 2001, *Cellulomonas cellulans *was reclassified as *Cellulosimicrobium cellulans*, and older clinical literature may therefore report the same organism as *Oerskovia xanthineolytica* [1].

Human infection is uncommon but clinically relevant. The organism has been recovered from blood, cardiac valves, peritoneal fluid, synovial or deep tissue, ocular specimens, cerebrospinal fluid, and other normally sterile sites. Most invasive infections have occurred in patients with central venous catheters, prosthetic valves, dialysis catheters, malignancy, chemotherapy exposure, renal replacement therapy, or other foreign material [2,3].

Awareness of this uncommon pathogen is becoming increasingly important because the population of patients living with long-term intravascular devices, prostheses, malignancy, transplantation, and other immunocompromising conditions continues to expand. At the same time, wider availability of matrix-assisted laser desorption ionization-time of flight mass spectrometry (MALDI-TOF MS), 16S ribosomal RNA (rRNA) gene sequencing, and other molecular methods is increasing recognition of isolates that would previously have been reported only as diphtheroids or coryneform bacilli [2-5].

Early diagnosis and appropriate treatment are clinically important. Failure to recognize repeated or deep-site isolation as true infection may delay repeat cultures, echocardiography, targeted antimicrobial therapy, and source control. Conversely, unnecessary prolonged treatment should be avoided when a single isolate is unsupported by compatible clinical evidence. This review therefore provides a clinically oriented synthesis of the published evidence, with particular attention to diagnostic significance, identification methods, antimicrobial susceptibility, treatment, and device-related source control.

## Review

Literature search strategy and review approach

This article was designed as a narrative review rather than a systematic review or meta-analysis. PubMed/MEDLINE was used as the principal bibliographic database, and the search was updated through July 23, 2026. Targeted searches of publisher websites and the reference lists of relevant articles and reviews were used to identify additional reports. The core search combined the terms “Cellulosimicrobium,” “Oerskovia,” “Cellulosimicrobium cellulans,” “Cellulosimicrobium funkei,” “Oerskovia xanthineolytica,” and “Oerskovia turbata” with clinical terms including “infection,” “bacteremia” or “bacteraemia,” “endocarditis,” “peritonitis,” “osteomyelitis,” “septic arthritis,” “endophthalmitis,” “meningitis,” “catheter,” “prosthesis,” “antimicrobial susceptibility,” and “treatment.”

Eligible publications were human case reports, case series, and reviews that provided clinically relevant information on infection site, host factors, microbiological identification, antimicrobial susceptibility, treatment, source control, or outcome. Environmental, industrial, veterinary, and nonhuman studies were excluded unless they directly informed taxonomy or laboratory identification. Duplicate publications, reports without sufficient clinical information, and articles in which *Cellulosimicrobium* could not be linked to a human clinical syndrome were excluded. Titles and abstracts were screened for relevance, followed by review of the available full text and reference lists.

Because the evidence consists predominantly of heterogeneous single-patient reports and small series, findings were synthesized narratively. No meta-analysis, meta-regression, effect estimate, confidence interval, p-value, or hypothesis test was calculated. The numerical frequencies cited in the manuscript are descriptive values reproduced from the most comprehensive published synthesis, which included 40 patients from 38 studies [4], and are not the result of a new pooled analysis. More recent reports, including a 2024 meningitis case, a 2025 endocarditis case, and a 2025 report of concomitant neck and lung masses, are discussed separately and were not added to those denominators [6-9].

A formal risk-of-bias score and duplicate independent data extraction were not undertaken because the article was conceived as a clinically oriented narrative review. Nevertheless, the interpretation explicitly considered the principal limitations of the underlying evidence, including incomplete species confirmation, heterogeneous susceptibility methods, selective publication of severe or unusual cases, missing follow-up, and the absence of prospective comparative studies. These limitations preclude population-level incidence estimates and definitive organism-specific treatment recommendations.

Taxonomy and microbiological characteristics

*Cellulosimicrobium cellulans* is the species most frequently implicated in human infection, including isolates described under the former name *O. xanthineolytica*. *Cellulosimicrobium funkei*, which incorporates clinical isolates previously classified as *Oerskovia turbata*, has been reported less often [1,4,5]. Species differentiation can be difficult because conventional biochemical profiles overlap and partial 16S rRNA gene sequences may not reliably distinguish *C. cellulans* from *C. funkei* [5].

On Gram staining, isolates may appear as pleomorphic, beaded, or branching Gram-positive rods that become shorter or coccobacillary with prolonged incubation. Colonies have been described as small, smooth, nonhemolytic, and yellow-pigmented, and isolates are commonly catalase-positive [5,7]. These features overlap with other coryneform and actinomycete-like organisms and can contribute to preliminary misclassification.

The mechanisms underlying pathogenicity are incompletely defined. However, the repeated association with intravascular catheters, prosthetic valves, dialysis catheters, and retained traumatic material indicates a capacity for persistence on foreign surfaces. Although dedicated biofilm studies in clinical isolates are limited, recurrence or persistence in device-associated cases supports early evaluation of implanted material as a potential reservoir [3,7-10].

Epidemiology and predisposing conditions

The 2024 narrative review identified 40 patients reported in 38 publications. The median age was 52.5 years, and 55% were male [4]. The predominance of reports from Europe and North America may reflect access to MALDI-TOF MS and molecular identification rather than the true geographic distribution of infection [4].

Important host and healthcare-associated factors were common. A central venous catheter was present in 27.5% of all reported patients and in 50% of patients with bacteremia. End-stage renal disease was reported in 15%, peritoneal dialysis in 12.5%, active malignancy in 15%, and a prosthetic cardiac valve in 10% [4]. Recent antimicrobial exposure, chemotherapy, neutropenia, previous surgery, and penetrating trauma were also repeatedly described [3,4,8,11-15]. These findings support an opportunistic and device-associated profile, but infection is not restricted to profoundly immunocompromised hosts. *Cellulosimicrobium funkei* bacteremia with probable prosthetic valve endocarditis,* C. cellulans* meningitis in an immunocompetent adult, and more recent endocarditis or unusual mass-forming presentations illustrate that clinically significant infection may occur outside the classic risk profile [5,6,16,17].

Clinical spectrum

The principal syndromes and practical implications are summarized in Table 1. Clinical categories may overlap, particularly when bacteremia accompanies infective endocarditis, osteoarticular infection, or another deep focus. The table is intended as a clinical guide rather than a quantitative summary.

**Table 1:** Clinical presentations, diagnostic clues, and management considerations in human Cellulosimicrobium infections Summarized are recurring clinical patterns from published case reports, small case series, and narrative reviews [3,4,6-17]. This summary is not a treatment guideline, and management should be individualized

Clinical presentation	Typical context and diagnostic clues	Main management considerations
Bloodstream infection and catheter-related bacteremia	Repeated positive blood cultures; concordant catheter and peripheral cultures; relapse after apparently adequate therapy; central venous or dialysis catheter.	Repeat cultures, minimal inhibitory concentration (MIC)-based therapy, evaluation for metastatic infection, and early consideration of catheter removal when bacteremia persists or recurs.
Infective endocarditis	Persistent or recurrent bacteremia; prosthetic valve or cardiac device; embolic events, heart failure, or paravalvular complications; transthoracic echocardiography may be nondiagnostic.	Transesophageal echocardiography when suspicion remains high; prolonged targeted therapy; early multidisciplinary evaluation for device extraction or valve surgery.
Osteoarticular, soft-tissue, and traumatic infection	Prosthetic joint, penetrating trauma, retained material, tenosynovitis, septic arthritis, or deep-tissue isolation.	Drainage or debridement, removal of infected or retained material when feasible, and prolonged therapy guided by site and susceptibility.
Peritoneal dialysis-associated peritonitis	Cloudy effluent, abdominal pain, positive peritoneal-fluid culture, and failure or relapse despite intraperitoneal treatment.	Appropriate intraperitoneal/systemic therapy and reassessment of the dialysis catheter; removal may be required to achieve cure or preserve safety.
Ocular and central nervous system infection	Ocular surgery or penetrating trauma for endophthalmitis; cerebrospinal-fluid or tissue isolation for meningitis or intracranial infection; infection may occur without classic immunosuppression.	Urgent site-specific management, including vitrectomy when indicated; CNS-penetrating therapy; and confirmation that the isolate is clinically concordant.
Other or atypical presentations	Respiratory, urinary, cutaneous, or mass-forming lesions are uncommon; specimen quality and histopathological or radiological concordance are essential.	Exclude colonization or contamination, seek a deep focus, and individualize treatment according to diagnostic certainty and source-control options.

Bloodstream Infection and Catheter-Related Bacteremia

Bloodstream infection is the most frequently reported presentation. In the 2024 synthesis, 22 of 40 patients had bacteremia, and half of these patients had a central venous catheter [4]. Repeated positivity in multiple blood culture sets, concordant peripheral and catheter cultures, recurrence, or a compatible septic syndrome strongly favors true infection rather than contamination [3,7,8,10,14].

Catheter salvage has occasionally succeeded, including a pediatric case treated with vancomycin and rifampicin [7]. However, other reports describe clearance only after line removal or late recurrence when a device-related reservoir persisted [8,14]. Retention should therefore be restricted to carefully selected patients with rapid microbiological clearance, no metastatic focus, and close follow-up.

Infective Endocarditis

Infective endocarditis is the most severe recognized manifestation. Eight cases were included in the 2024 review; half involved prosthetic valves, embolic complications occurred in 37.5%, paravalvular abscesses in 25%, and mortality reached 62.5% [4]. These proportions must be interpreted cautiously because they derive from a very small and highly selected case-report population.

Initial blood cultures may be interpreted as contaminants, transthoracic echocardiography may be nondiagnostic, and disease may remain indolent before heart failure, embolic phenomena, cerebral infection, or paravalvular complications become apparent [3,5,9,10]. A 2025 report described a subacute infection detected after tricuspid valve replacement and pacemaker extraction despite repeatedly negative preoperative cultures, supporting culture and molecular testing of explanted material when suspicion persists [6]. Persistent or recurrent bacteremia, prosthetic cardiac material, unexplained systemic inflammation, or embolic manifestations should prompt transesophageal echocardiography and early collaboration among infectious disease, cardiology, cardiac surgery, and microbiology teams [3,6,9,10].

Osteoarticular, Peritoneal, Ocular, and Other Infections

Osteoarticular infections include prosthetic joint infection, tenosynovitis, septic arthritis, and deep infection after penetrating trauma. They commonly require prolonged antimicrobial therapy combined with drainage, debridement, or removal of retained material [4,11]. All five peritoneal dialysis-associated peritonitis cases in the 2024 review occurred in dialysis patients; survival was favorable, but technique failure and conversion to hemodialysis occurred [4,12].

Endophthalmitis has followed ocular surgery or penetrating trauma and frequently required vitrectomy in addition to antimicrobial treatment [4,13]. Central nervous system disease remains rare. A 2024 report described culture-confirmed *C. cellulans *meningitis in an immunocompetent adult who improved with ceftriaxone, emphasizing that a compatible sterile-site isolate should not be dismissed solely because classical risk factors are absent [16]. A 2025 report of concomitant neck and lung masses after a dental procedure further broadened the spectrum of potential presentations, although causal attribution in unusual syndromes requires careful clinicopathological correlation [17].

Diagnostic interpretation

When Should the Isolate Be Considered Clinically Significant?

A Cellulosimicrobium isolate should not be dismissed automatically as a contaminant. Features supporting clinical significance include recovery from multiple blood culture sets, persistent or recurrent positivity, isolation from a normally sterile site, growth from deep tissue or explanted material, compatible local or systemic signs, and the presence of a catheter, prosthesis, shunt, or retained foreign body [3,4,7-10,16]. The probability of true infection is further increased when no alternative pathogen or diagnosis explains the syndrome.

Conversely, a single positive bottle in a clinically stable patient without a device or compatible focus may represent contamination. Repeat cultures and clinical reassessment are preferable to either immediate dismissal or prolonged empirical treatment. Communication between the laboratory and clinical team is essential because the organism may initially be reported only as a coryneform Gram-positive rod.

Laboratory Identification

Conventional phenotypic systems may identify an isolate only as a coryneform organism or may misidentify it because of incomplete databases and overlap with related taxa [2,3,5]. The MALDI-TOF MS provides rapid identification and has increased species-level recognition, but results should be interpreted in relation to database coverage, confidence score, colony age, and extraction method [2,3].

Sequencing of the 16S rRNA gene is useful when MALDI-TOF MS is unavailable, yields a low-confidence result, or conflicts with phenotypic findings. Partial 16S sequences may not reliably differentiate *C. cellulans* from *C. funkei*, and additional phenotypic or reference-laboratory analysis may be required [5]. In culture-negative or diagnostically complex infections, broad-range or next-generation sequencing can identify the organism from tissue or other sterile material, but molecular results must remain clinically contextualized [6,10]. For suspected invasive disease, laboratories should retain the isolate, document the identification platform and confidence score, and consider confirmatory testing. Reporting only 'diphtheroids' or 'coryneform bacilli' may obscure an important pathogen when the same organism is recovered repeatedly or from a sterile site.

Antimicrobial Susceptibility Testing

Susceptibility data are heterogeneous because published reports used broth or agar dilution, disk diffusion, gradient diffusion, and different interpretive criteria. In the largest synthesis, reported resistance was 70% to clindamycin, 60% to penicillin, 53.3% to aminopenicillins, 42.9% to cephalosporins, 33.3% to aminoglycosides, 29.4% to tetracyclines, and 16.7% to carbapenems. Resistance was lower for trimethoprim-sulfamethoxazole at 8.7%, and no vancomycin-resistant isolate was reported among 29 tested patients [4]. These values are descriptive and should not be interpreted as population-based resistance rates.

Because species-specific clinical breakpoints and robust outcome correlations are lacking, minimum inhibitory concentration (MIC) testing is preferable for invasive infection whenever feasible. The Clinical and Laboratory Standards Institute (CLSI) M45 provides methodological and interpretive guidance for infrequently isolated or fastidious bacteria when susceptibility cannot be predicted from identification alone [15]. Reports should specify the method, MIC values, and interpretive framework, particularly when criteria are extrapolated from related Gram-positive rods. The available evidence does not support empirical beta-lactam monotherapy for severe infection. Vancomycin is the most consistently active initial agent in published experience, while trimethoprim-sulfamethoxazole, linezolid, rifampicin, carbapenems, or other agents should be considered only after review of isolate-specific susceptibility, infection site, drug penetration, interactions, toxicity, and host factors [3,4,7,16].

Therapeutic Management and Source Control

No randomized trial or pathogen-specific treatment guideline is available. Therapy should be individualized according to syndrome, severity, susceptibility results, organ function, drug penetration, and the feasibility of source control. For endocarditis, CNS infection, prosthetic joint infection, or other deep-seated disease, treatment duration should follow established principles for the anatomical syndrome while being adjusted to microbiological response and surgical findings.

Rifampicin has been included in some combinations for catheter-associated or foreign-body infection, but evidence is limited to individual reports [7]. It should not be used alone because resistance can emerge rapidly. Combination treatment cannot be recommended routinely and should be reserved for selected severe device-associated infections after specialist review of MIC results and potential toxicity.

Source control is a recurring determinant of success. Reported interventions include removal of central venous or dialysis catheters, excision of retained foreign bodies, drainage and debridement, vitrectomy, prosthetic joint removal, cardiac device extraction, and valve surgery [3,6,8,9,11-14]. Persistent bacteremia, relapse, mechanical complications, or poor clinical response should trigger renewed investigation for an uncontrolled focus.

Outcomes and Prognosis

Across the 40 patients included in the 2024 review, all-cause mortality was 20% and infection-attributable mortality was 17.5% [4]. Mortality was concentrated in bacteremia and infective endocarditis: 36.4% of patients with bacteremia and 62.5% of patients with endocarditis died [4]. In contrast, no deaths were reported among the small groups with osteoarticular infection, peritoneal dialysis-associated peritonitis, or endophthalmitis, although visual impairment, prolonged therapy, surgery, or loss of dialysis technique occurred [4,13].

These estimates are vulnerable to publication bias, small denominators, selective reporting, and confounding by age, malignancy, organ failure, and prosthetic infection. They should not be interpreted as stable prognostic rates. Nevertheless, the severe course of reported endocarditis supports early echocardiographic assessment, multidisciplinary management, and timely surgical evaluation when indicated.

Practical Implications for Clinicians and Laboratories

A practical approach begins with verification of specimen quality and review of the number and timing of positive cultures. When the organism is recovered repeatedly or from a sterile site, the laboratory should pursue species-level identification by MALDI-TOF MS and confirm uncertain results by 16S rRNA sequencing or a reference laboratory. The clinical team should obtain repeat blood cultures, examine implanted devices, investigate a deep focus, and request echocardiography when bacteremia persists or cardiac prosthetic material is present.

Empirical therapy for severe infection should include an agent with reliable Gram-positive activity, most commonly vancomycin, until MIC data are available. Definitive therapy should be narrowed according to susceptibility, site, and patient factors. Device retention should not be the default strategy: removal or surgical intervention should be discussed early in recurrent bacteremia, endocarditis, prosthetic joint infection, and dialysis-associated peritonitis.

Limitations

This review is limited by the rarity of the organism and the predominance of case reports. Many publications lack complete information on identification methods, susceptibility testing, MIC values, source control, treatment duration, or long-term follow-up. The numerical frequencies were taken from a previously published 40-patient synthesis and were not recalculated through a new pooled analysis. Newer cases were discussed qualitatively rather than incorporated into historical denominators.

Because this is a narrative review, a PRISMA flow diagram, formal risk-of-bias scoring, and duplicate independent data extraction were not performed. Publication bias likely favors severe, unusual, or successfully identified cases, whereas infections in settings without MALDI-TOF MS or molecular methods may remain unrecognized. The available evidence therefore supports pragmatic clinical principles but not definitive incidence estimates, comparative effectiveness conclusions, or organism-specific treatment guidelines.

## Conclusions

Human infections caused by *Cellulosimicrobium *species are rare but may be severe, particularly when bloodstream infection or infective endocarditis occurs. The organism should not be dismissed as contamination when it is recovered repeatedly, isolated from a sterile or deep site, or detected in a patient with prosthetic material, an intravascular catheter, dialysis access, compatible clinical findings, or immunosuppression. The absence of classic risk factors does not exclude true infection.

Optimal management requires close collaboration between clinicians and the microbiology laboratory. Species-level identification, MIC-based susceptibility testing, repeat cultures, investigation for a deep or device-associated focus, appropriate antimicrobial therapy, and timely source control are central. Future reports should provide detailed identification data, MIC values, device-management decisions, treatment duration, and long-term outcomes to strengthen the evidence base for this uncommon pathogen.

## References

[1] Schumann P, Weiss N, Stackebrandt E (2001). Reclassification of Cellulomonas cellulans (Stackebrandt and Keddie 1986) as Cellulosimicrobium cellulans gen. nov., comb. nov. Int J Syst Evol Microbiol.

[2] Tekippe EM, Shuey S, Winkler DW, Butler MA, Burnham CA (2013). Optimizing identification of clinically relevant Gram-positive organisms by use of the Bruker Biotyper matrix-assisted laser desorption ionization-time of flight mass spectrometry system. J Clin Microbiol.

[3] Rivero M, Alonso J, Ramón MF (2019). Infections due to Cellulosimicrobium species: case report and literature review. BMC Infect Dis.

[4] Ioannou P, Vorria A, Samonis G (2024). Cellulosimicrobium infections in humans — a narrative review. Antibiotics.

[5] Petkar H, Li A, Bunce N, Duffy K, Malnick H, Shah JJ (2011). Cellulosimicrobium funkei: first report of infection in a nonimmunocompromised patient and useful phenotypic tests for differentiation from Cellulosimicrobium cellulans and Cellulosimicrobium terreum. J Clin Microbiol.

[6] Mueller TT, Steffen J, Scherer C, Angstwurm MW (2025). Cellulosimicrobium cellulans endocarditis: a challenge for detection and treatment in interdisciplinary teams. Infection.

[7] Rowlinson MC, Bruckner DA, Hinnebusch C, Nielsen K, Deville JG (2006). Clearance of Cellulosimicrobium cellulans bacteremia in a child without central venous catheter removal. J Clin Microbiol.

[8] Zamora JAG, Camps N (2018). Bacteremia caused by Cellulosimicrobium in a bone marrow transplant patient: a case report and literature review. IDCases.

[9] Monticelli J, Gerloni R, Farina C, Knezevich A, Dore F, Luzzati R (2019). Cellulosimicrobium cellulans aortic prosthetic valve endocarditis. Access Microbiol.

[10] Zhang H, He C, Tian R, Wang R (2020). A case report of the differential diagnosis of Cellulosimicrobium cellulans-infected endocarditis combined with intracranial infection by conventional blood culture and second-generation sequencing. BMC Infect Dis.

[11] Tucker JD, Montecino R, Winograd JM, Ferraro M, Michelow IC (2008). Pyogenic flexor tenosynovitis associated with Cellulosimicrobium cellulans. J Clin Microbiol.

[12] Sug Kim J, Won Lee T, Gyoo Ihm C, Jin Kim Y, Mi Moon S, Joo Lee H, Hwan Jeong K (2015). CAPD peritonitis caused by co-infection with Cellulosimicrobium cellulans (Oerskovia xanthineolytica) and Enterobacter cloacae: a case report and literature review. Intern Med.

[13] Jaru-Ampornpan P, Agarwal A, Midha NK, Kim SJ (2011). Traumatic Endophthalmitis due to Cellulosimicrobium cellulans. Case Rep Ophthalmol Med.

[14] Coletta-Griborio E, Portela GR, García JMN, Bratos-Pérez MÁ (2017). Bacteremia due to Cellulosimicrobium cellulans associated with central catheter for hemodialysis. Enferm Infecc Microbiol Clin.

[15] (2016). M45 | Methods for Antimicrobial Dilution and Disk Susceptibility Testing of Infrequently Isolated or Fastidious Bacteria. https://clsi.org/shop/standards/m45/.

[16] Narayan P, Duble S, Shetty A, Sekhar M, Shetty R, Govindarajan SK (2024). A rare case of meningitis: can Cellulosimicrobium cellulans cause meningitis in a non-immunocompromised person?. Cureus.

[17] Sheehan KM, Moloney G, Murphy O (2025). Concomitant neck and lung masses post dental procedure-a potential novel presentation of the Cellulosimicrobium species in humans. Infect Dis Rep.

